# Efficacy of Adenosine Versus Verapamil in Terminating Acute Attacks of Paroxysmal Supraventricular Tachycardia: A Systematic Review

**DOI:** 10.7759/cureus.45946

**Published:** 2023-09-25

**Authors:** Vamsi Krishna Pachchipulusu, Yubraj Paudel, Anandkumar Patel, Beenish Najam, Heet N Desai, Janan Illango, Kofi D Seffah, Mahendar Kumar, Namballa Naveen, Safeera Khan

**Affiliations:** 1 Internal Medicine, California Institute of Behavioral Neurosciences & Psychology, Fairfield, USA; 2 Neurology, Shalby Hospitals Naroda, Ahmedabad, IND; 3 Medicine, Maharshi Hospital Private Limited, Surendranagar, IND; 4 Research, California Institute of Behavioral Neurosciences & Psychology, Fairfield, USA; 5 Internal Medicine, Piedmont Athens Regional Medical, Athens, USA; 6 Anaesthesia, Royal College of Surgeons in Ireland, Drogheda, IRL; 7 Internal Medicine, Steel Authority of India (SAIL) Hospital, Dhanbad, IND

**Keywords:** efficacy, arrhythmia, paroxysmal supraventricular tachycardia, verapamil, adenosine

## Abstract

Paroxysmal supraventricular arrhythmias are a group of common rhythm disturbances that are often prevalent, frequently recurrent, sporadic, and life-threatening. These arrhythmias are precipitated by factors such as age, sex, and associated comorbidities. Typically, patients with paroxysmal arrhythmias are asymptomatic during evaluation, and the condition is often detected incidentally. Symptoms associated with these arrhythmias include palpitations, fatigue, light-headedness, chest discomfort, dyspnea, presyncope, and, less commonly, polyuria and serious psychological distress. In terms of treatment, common modalities include antiarrhythmic drug therapy and catheter ablation. When selecting drug therapy, factors such as comorbidities, patient-specific modifiers, preferences, follow-up frequency, and cost-effectiveness are taken into account. For long-term treatment, calcium channel blockers are often used instead of adenosine, while adenosine is preferred for acute attacks due to its higher efficacy. Comparatively, adenosine and verapamil are commonly used drugs in the emergency setting to treat paroxysmal supraventricular tachycardia (PSVT). Adenosine exhibits a faster onset of action, but adverse effects occur more commonly, whereas verapamil has a slower onset of action and a lower incidence of adverse effects. We searched for articles from PubMed, PubMed Central (PMC), and Science Direct, and these articles were reviewed systematically. After applying the search strategy to these databases, 195 articles were identified. Fourteen of these were finalized for review. The efficacy of adenosine versus verapamil in terminating acute attacks of PSVT is reviewed in our article.

## Introduction and background

**Figure 1:**
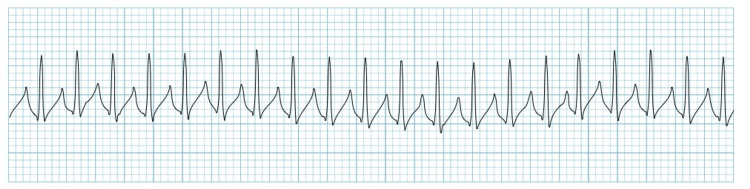
Paroxysmal supraventricular tachycardia (PSVT)

Sudden cardiac death is the most feared consequence of paroxysmal supraventricular tachycardia (PSVT). According to the American Heart Association (AHA), almost half of cardiac deaths are due to PSVT, accounting for almost 550,000 annually, which is a striking number. Females are two times more affected compared to males [1,2]. PSVT is a preventable cause of death that can be avoided by using an implantable cardiac defibrillator (ICD) or anti-arrhythmic drugs such as adenosine, calcium channel blockers (CCBs), beta-blockers, amiodarone, or digoxin. While catheter ablation is another procedure that destroys abnormal cells responsible for abnormal heart rhythms caused by PSVT, it is highly successful and curative. Intervention signs of congestive heart failure usually develop if tachycardia sustains for six to 12 hours [3,4]. Electrocardiogram of PSVT is illustrated below in Figure 1.

**Figure 2:**
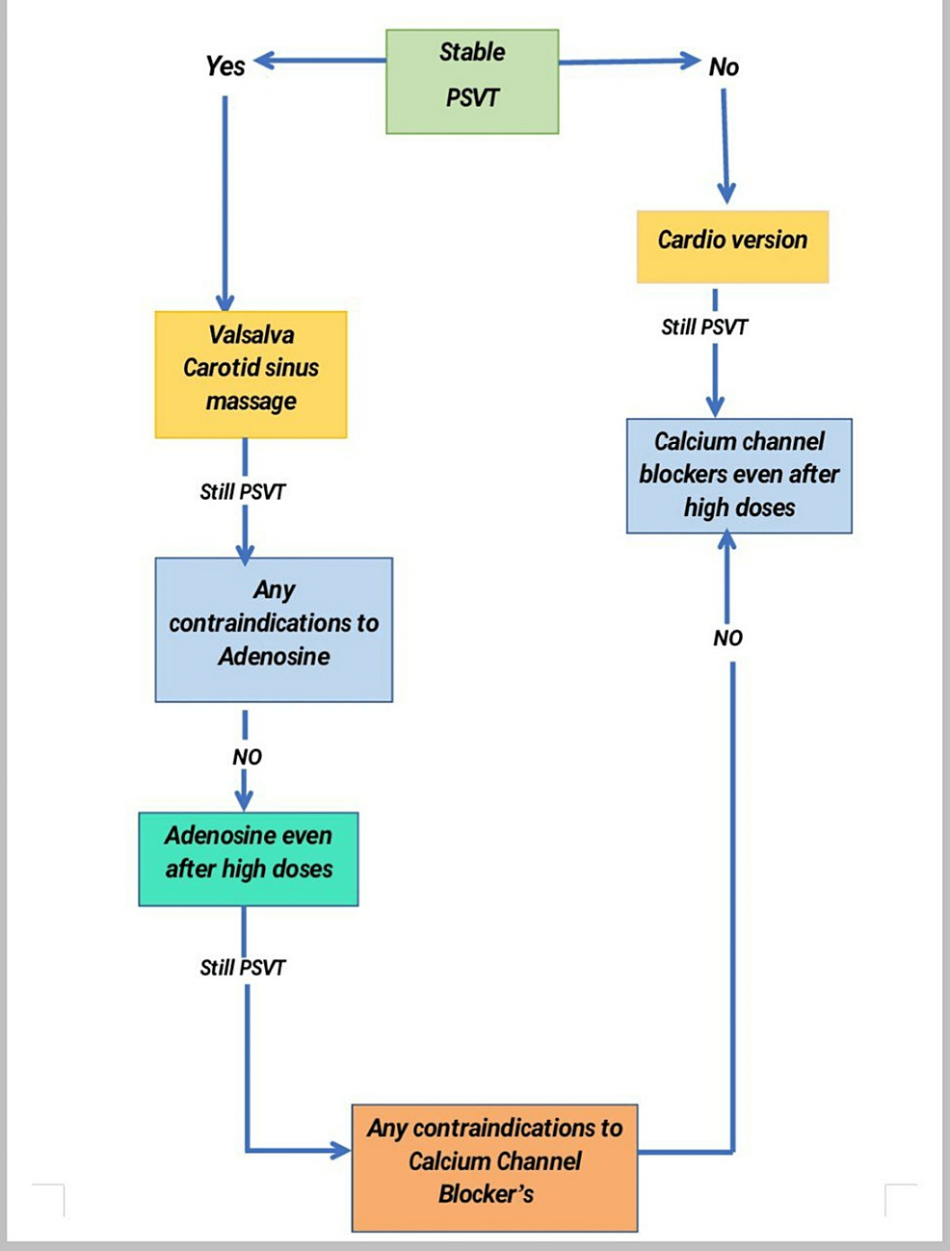
Protocol for Treatment of Paroxysmal Supraventricular Tachycardia (PSVT)

The initiation of a re-entry circuit via the atrioventricular (AV) node causes PSVT. With a QRS interval of 100 milliseconds or less on an electrocardiogram (ECG), supraventricular tachycardia is typically a narrow complex tachycardia. Some of the vagal techniques like the Valsalva maneuver, carotid artery massage, forced breath holding, and facial immersion in cold water can be done initially in hemodynamically stable patients with continuous ECG monitoring and surprisingly these methods can terminate the episode in 20-25% of cases [5]. The treatment options for PSVT are seen in Figure 2.

## Review

Methods 

We included studies where adenosine or verapamil was used for PSVT treatment in all age groups. We used databases like PubMed, PubMed Central (PMC), Medline, Cochrane Library, Scopus, and MDPI. 

The Preferred Reporting Items for Systematic Review and Meta-Analyses (PRISMA) 2020 standards were developed to make reporting more thorough and transparent. These were followed throughout the entire investigation.

Search Study 

We have created a Medical Subject Heading (MeSH) technique for PubMed using Boolean AND OR ( "Tachycardia, Supraventricular/drug therapy"[Majr]OR "Tachycardia, Supraventricular/therapy"[Majr] ) AND ( "Adenosine/adverse effects"[Mesh]A PRISMA flow chart presenting the selection of articles is shown in Figure OR "Adenosine/therapeutic use"[Mesh] OR "Adenosine/toxicity"[Mesh] )AND( "Verapamil/adverse effects"[Mesh] OR "Verapamil/therapeutic use"[Mesh] OR "Verapamil/toxicity"[Mesh] ).

The majority of the articles that met the inclusion and exclusion criteria were evaluated as being significant to the search query. Table 1 lists the inclusion and exclusion criteria. Different criteria were used to evaluate qualitative research, therapy, and results in each clinical trial.

**Table 1:** The inclusion and exclusion criteria PSVT= paroxysmal supraventricular tachycardia

INCLUSION CRITERIA	EXCLUSION CRITERIA
Papers written and published in English language	Grey literature
Papers focusing on adenosine and verapamil used for PSVT treatment.	Papers focusing on neonates and pregnant age groups
Papers focusing on all age groups	

We combined the articles in an Excel sheet for duplicate removal. The records have been initially reviewed based on titles and abstracts, and irrelevant articles have been excluded, followed by a review of full-text articles. The 2015 American College of Cardiology/American Heart Association/Heart Rhythm Society guideline on the therapy of adults with supraventricular tachycardia was among the clinical practice guidelines we included. We also included randomised controlled trials, review articles and other relevant literature. Additionally, we manually retrieved papers from pertinent articles' reference lists. 

Results

We examined PubMed, PMC, Medline, Cochrane Library, Scopus, and MDPI. A total of 255 articles were discovered after combining 165 in PubMed with 82 Cochrane Library articles and eight MDPI articles. Excel was used to remove 42 duplicate articles, and 18 articles were marked as ineligible by automation tools, leaving 195 items in total for screening. Sixty-four articles were removed after the title and abstract screening and 86 articles were not retrieved. Thirty-one out of 45 publications were disqualified for the reasons mentioned in the PRISMA chart after being assessed for eligibility. Following the screening, 14 papers compared the effectiveness of adenosine versus verapamil in treating PSVT. In Tables 2, 3, 4 we have described the quality appraisal tool for studies that were included in the systematic review.

**Table 2:** Summary of randomised controlled trials using the Cochrane assessment tool

Cochrane appraisal	Year of study	Random sequence generation	Allocation concealment	Blinding of participants & personnel	Blinding of outcome assessment	Incomplete outcome data
Di Marco et al. [6]	1990	Yes	yes	yes	no	No
Lim et al. [7]	2009	Yes	yes	yes	yes	Yes
Stambler et al. [8]	2022	Yes	no	yes	yes	No

**Table 3:** Amstar Checklist for Systematic Review

Amstar checklist	Ahmad et al.	Alabed et al. 2017	Delaney et al. 2011
1.was an “a priori design provided”	yes	yes	yes
2.was there any duplicate study selection and data extraction	yes	yes	yes
3.was a comprehensive literature search performed	yes	yes	yes
4.was the status of publication i.e. grey literature used as an inclusion criteria	No	No	No
5.was the list of studies (included and excluded provided)	yes	yes	yes
6.were the characteristics of included studies provided	yes	yes	yes
7.was the scientific quality of included studies assessed and documented	yes	yes	yes
8.was the scientific quality of included studies used appropriately in formulating conclusions	yes	yes	yes
9.were the methods used to combine the findings of studies appropriate	yes	yes	yes
10.was the likelihood of publication bias assessed	yes	unclear	unclear
11.was the conflict of interest stated	yes	yes	yes
Total scores	10	8	9

**Table 4:** JBI Checklist

JBI checklist questions	Siddhartha et al.
Were the patient’s demographic characteristics clearly described	yes
Was the patient's history clearly described and presented as a timeline	yes
Was the current clinical condition of the patient on presentation clearly described	yes
Were the diagnostic tests or assessment methods and the results clearly described	no
Was the intervention and treatment procedures clearly described	yes
Was the post-intervention clinical condition clearly described	yes
Were adverse events or unanticipated events identified and described	yes
Does the case report provide takeaway lessons	yes

Different criteria were used to evaluate qualitative research, therapy, and results in each clinical trial. The clinical studies that we included in the article are summarised in Table 5.

**Table 5:** Summary of clinical trials PSVT: paroxysmal supraventricular tachycardia, CCBs: calcium channel blockers, RCT: randomized controlled trial

Author & year of publication	Intervention Studied	Type of study	No. of patients	Conclusion
Bibas et al. 2016 [9]	Diagnosis and management of supraventricular tachycardia	Retrospective cohort study	1754	Curative treatment is radiotherapy
Steinbeck et al. 2004 [2]	Preferential use of adenosine or verapamil in treatment of PSVT	Retrospective observational study	106	The rate of tachycardia is used to determine which drugs are effective.
Althunayyan et al. 2018 [10]	Adenosine response among numerous variables in PSVT patients	Retrospective case-control study	38	Difference in response to adenosine among groups was attributed to the presence of heterogeneous conducting pathways
Ballo et al. 2004 [11]	Heart rate may affect the efficacy of adenosine, verapamil and carotid sinus massage in terminating symptomatic episodes of PSVT	Prospective cohort study	175	Heart rate predicts sinus rhythm restoration in adult patients experiencing symptomatic PSVT bouts
Lee Lin et al. 1997 [12]	Long-term efficacy of slow pathway catheter ablation in patients with spontaneous supraventricular tachycardia and dual atrioventricular pathways without inducible tachychardia	Prospective cohort study	129	Slow pathway catheter ablation is beneficial in the elimination of PSVT recurrence
Prystowsky et al. 2003 [5]	Termination of PSVT by tecadenoson-a novel adenosine receptor agonist	Prospective cohort study	37	Tecadenoson suppressed AV nodal conduction to quickly end prolonged PSVT without inducing hypotension.
Di Marco et al. 1990 [6]	Assess the safety and efficacy of adenosine in terminating PSVT	Prospective randomised control trial	359	Adenosine's overall effectiveness is comparable to verapamil's, although its onset is rapid
Turkoglu et al. 2009 [13]	Wide QRS complexes observed during pharmacologic termination of supraventricular tachycardia	Prospective cohort study	74	Five different patterns of ventricular ectopy were observed during termination of tachycardia, most of them do not directly terminate tachycardia
Lim et al. 2009 [7]	Comparison of slow infusion of calcium channel blockers with intravenous adenosine in emergency treatment of supraventricular tachycardia	Randomised control trial	206	Slow infusion of calcium channel blockers is safe and affordable for healthcare systems where availability of adenosine is limited.
Ahmad et al. 2021 [14]	Compare the efficacy of adenosine and verapamil in the treatment of PSVT	Systematic review	3111	Adenosine is the first line treatment of PSVT, but both the drugs adenosine and CCBs have shown promising results regarding safety and efficacy.
Alabed et al. 2017 [15]	Compare effects of adenosine versus CCBs in terminating SVT	Systematic review	622	No differences in effects of adenosine and calcium channel antagonists for the treatment of SVT low-quality research suggests there are no discernible variations in the incidence of hypotension upon returning to sinus rhythm.
Delaney et al. 2011 [16]	Examine the adverse effects of adenosine and verapamil and their relative effectiveness.	Systematic review	8 trials	Adenosine and verapamil have similar efficacy in the treatment of PSVT. Minor adverse effects and overall adverse effects are higher with adenosine, whereas verapamil has a higher rate of hypotension
Stambler et al. 2022 [8]	Use of etripamil as a patient-administered option for PSVT termination outside of the health care setting.	Prospective RCT	180	Etripamil nasal spray has given promising results in changing treatment protocol for PSVT.

**Figure 3:**
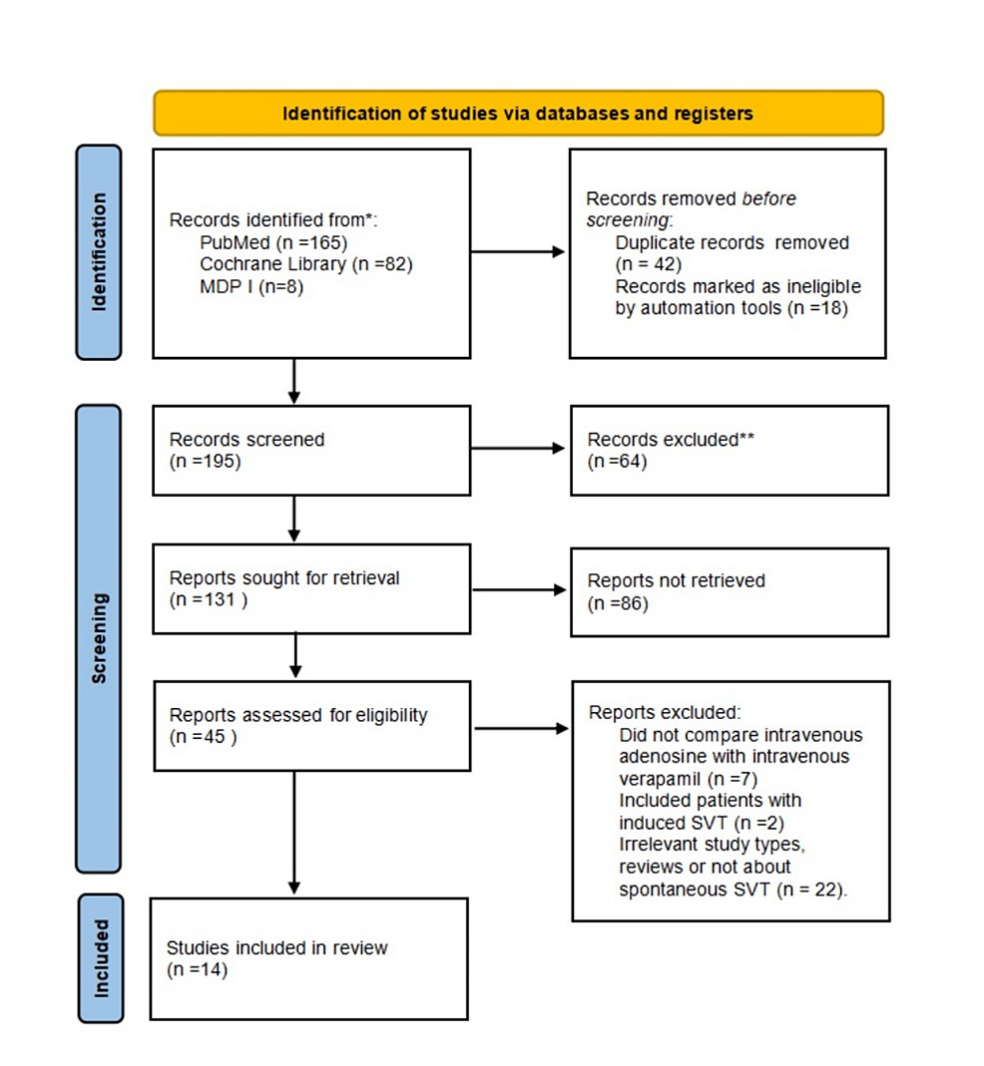
PRISMA flow diagram Adapted from source: Page et al. [17] PRISMA - Preferred Reporting Items for Systematic Review and Meta-Analyses

A PRISMA flow chart presenting the selection of articles is shown in Figure 3.

Given that both atrial fibrillation (AF) and PSVT lack P waves, it is simple but essential to distinguish between the two conditions. You can tell them apart without much difficulty because PSVT will have a regular R-R interval and AF will have an erratic R-R interval.

Discussion

We have conducted a systemic review to compare the effectiveness of adenosine versus verapamil in the treatment of PSVT. A variety of tachyarrhythmias known as paroxysmal supraventricular tachycardia originate from a circuit or focal point involving the atria or the atrioventricular node. The most frequent types of PSVT seen in primary care include atrioventricular nodal re-entrant tachycardia, atrioventricular re-entrant tachycardia, atrial tachycardia, and atrial flutter.

Patients with hemodynamically stable SVT should first have 12-lead ECG before attempting a diagnostic and therapeutic trial with vagal maneuvers or intravenous (IV) adenosine. Asymptomatic patients should be evaluated with an exercise stress test and ambulatory ECG monitoring and calcium channel blockers can be given as an outpatient treatment.

According to a retrospective case-control study involving 38 patients, non-pharmacological interventions were used initially in stable persons; if symptoms persist, IV adenosine at a dosage of 6 mg and a subsequent dose of 12 mg should be tried. A transient AV block may reveal atrial flutter or ectopic P waves, making IV adenosine a useful diagnostic tool. Adenosine administration to a patient should always be followed by ECG monitoring [7]. Adenosine is increasingly preferred over calcium-channel or beta-blockers, with the exception of people with severe asthma; while both adenosine and calcium-channel blockers like verapamil and diltiazem remain the go-to drugs. This is because of the rapid onset of action and very short half-life of adenosine compared to calcium channel blockers [3].

**Figure 4:**
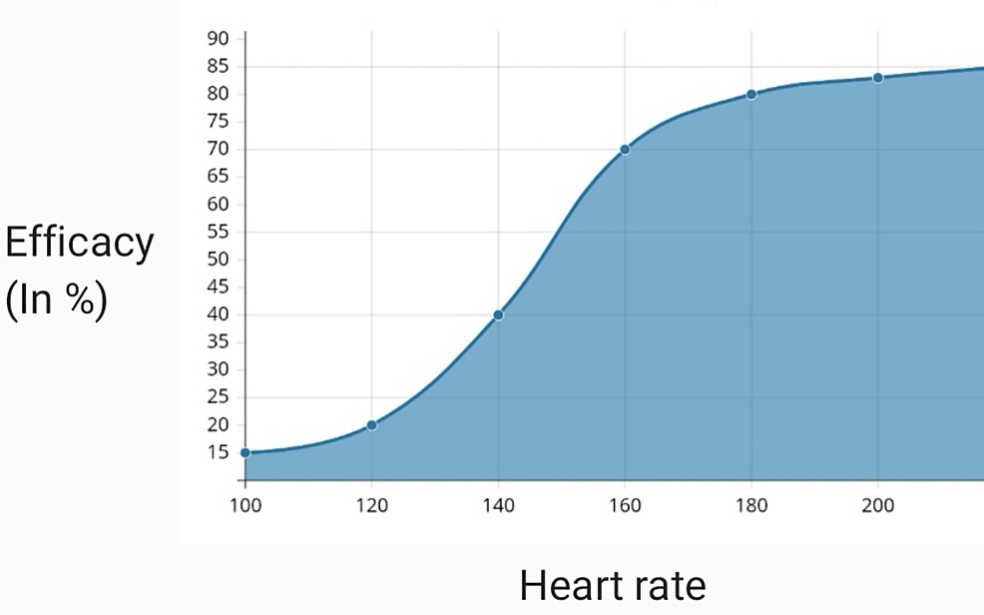
Adenosine

**Figure 5:**
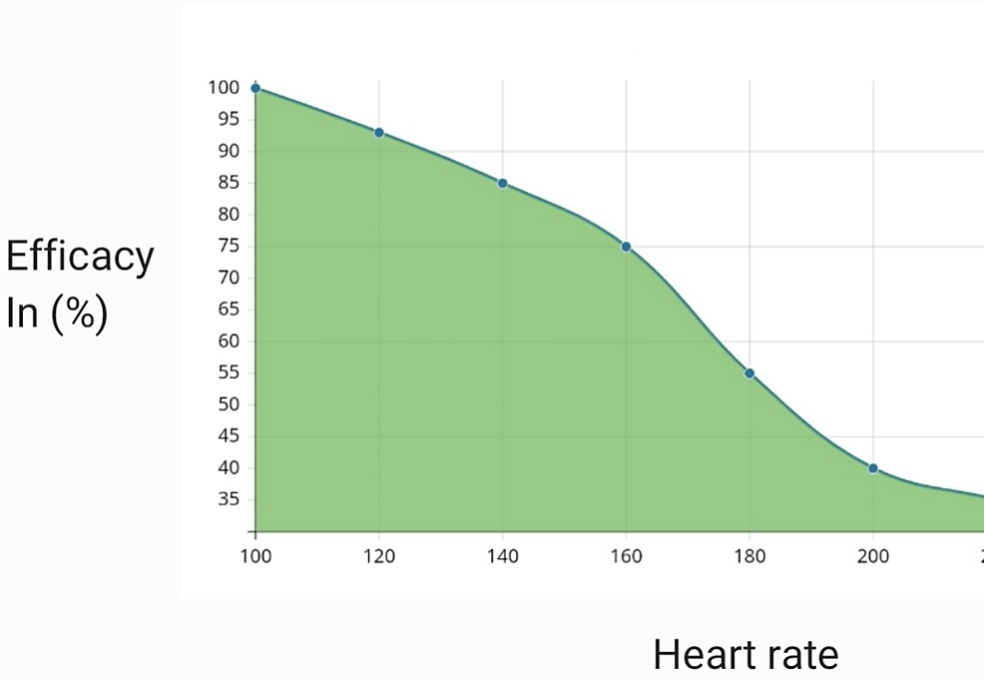
Verapamil

According to a prospective cohort study the efficacy of these drugs depends mainly on the rate of tachycardia. If the heart rate was more than 166 beats/min, adenosine was effective, whereas if the heart rate was less than 138 verapamil was effective. In establishing association between the increased heart rate and the likelihood that a carotid massage or drugs like adenosine, verapamil would be able to stop the arrhythmia in a group of 106 people, verapamil and adenosine both effectively stopped PSVT (74.4% and 81.8%, respectively), although carotid sinus massage had a much lower success rate (32.4%) [11]. Interestingly, the effectiveness of the two medications was inversely correlated with the rate of the tachycardia: after adenosine, the probability of termination of tachycardia was >75% for rates of tachycardia over 166 beats/min and rapidly declined at lower tachycardia rates down to 25% at a rate of 138 beats/min. On the other hand, the likelihood of tachycardia termination was >75% when verapamil was taken at heart rates under 186 beats per minute, however the probability of success fell more quickly (25% probability at 241 beats per minute). The impact of heart rate on verapamil effectiveness was somewhat less than the impact of tachycardia on adenosine effectiveness, which was the opposite. In the patient group receiving carotid sinus massage, there were no discernible impacts of tachycardia rates on the success of termination. According to a comparison of the two probability curves created in the adenosine and verapamil groups, adenosine seems to be more effective at treating tachycardias with rates of at least 173 beats/min, but verapamil may be more effective at treating lower rates of tachycardia [7]. The efficacy of adenosine at various heart rates is shown below in Figure 4, and the efficacy of verapamil at various heart rates is shown in Figure 5.

Mechanism of action of adenosine and verapamil

The major action of adenosine is to suppress the beta-adrenergic system by activating the K+ (potassium) current and inhibiting catecholamine-stimulated adenylate cyclase activity [6]. IV adenosine has a rapid onset of action compared to verapamil, but there is a high recurrence of arrhythmia compared to verapamil. According to a retrospective cohort study, adenosine sensitivity variations among PSVT patients were unrelated to their demographics, prior illnesses, and clinical characteristics. As a result, the heterozygosity in conducting routes may be responsible for the variation in adenosine response between groups. In comparison to the second bolus of adenosine, the first bolus exhibited higher sensitivity and specificity, and their ideal values were predicted by HR deceleration [9].

Tecadenoson (CVT-510) activates the A1Ado R selectively and prolongs atrioventricular nodal conduction at doses lower than those required to cause A2Ado R-mediated coronary and peripheral vasodilation, thus it is considered to be a novel adenosine receptor. Tecadenoson stops AV node-dependent supraventricular tachycardia without causing hypotension or broncho-constriction; in contrast, adenosine non-selectively stimulates all four Ado R subtypes and has undesirable side effects [5].

Verapamil exerts a blocking action on L-type calcium channels, thus preventing entry of slow inward calcium current due to its inhibiting effect on these calcium channels. Another mechanism that may be involved is the blocking of the rapid segment of the delayed rectifier potassium current. Thus, it caused the atrial effective refractory period to increase in healthy adults at low heart rates while shortening it at high heart rates, indicating a significant effect on the delay of rectifier potassium current at slow rates and the calcium current at higher rates [8]. Hypotensive episodes may occur with rapid bolus infusions of the calcium channel blocker. It is suggested to use slow infusions of calcium channel blockers; there has been no documentation of hypotension to any significant degree when a slow infusion of calcium channel blockers is used [12,15].

Adenosine's greater efficacy in treating very rapid supraventricular tachycardia may be due to its ability to effectively block adrenergic input, rather than verapamil's ineffectiveness. Adrenergic system activation is a key factor to block this type of tachycardia [8].

The type of PSVT that is commonly found in the general population is atrioventricular nodal reentrant tachycardia (AVNRT), this is seen commonly due to activation of both atria and ventricles at the same time. In the case of an AVNRT, P-waves are rarely seen on the electrocardiogram strip. In the inferior leads we can observe a pseudo-S' deflection in place of a P-wave, however, P-waves can be suppressed. While a pseudo-R-wave is seen in lead V1, the most critical ECG finding having a high sensitivity of diagnosis is a pseudo-R-wave, positive deflection at the end of the Q-wave, R-wave, and S-wave (QRS) complex during tachycardia seen on ECG but this deflection is generally not seen in a normal sinus rhythm [9]. According to a randomised control trial, it was shown that ventricular beats after SVT termination occurred in 27% of patients after verapamil administration and in 33% of patients after adenosine administration and these beats were thought to be the cause of the tachycardia's termination [13]. According to certain research, adenosine promotes refractoriness, decreases atrioventricular node conduction, and prevents sinus node automaticity, and some drug-induced ventricular arrhythmias.

Contraindications to Adenosine

When a patient has severe coronary artery disease, adenosine should be used with caution, since the vasodilation of normal coronary vessels might produce ischemia in a vulnerable territory, an attempt of giving adenosine in these patients can be only done when there is full resuscitative equipment available, immediate resuscitative measures can be done in case of adverse reaction to adenosine, there won't be any long term adverse effects to adenosine, most of the times reaction to adenosine are common but they last for a very brief period and are minor and concise [14]. Conditions where it can be safely used are in patients without any prior heart disease and young patients who can tolerate the side effects compared to elder patients.

Contraindications to Verapamil

Verapamil is contraindicated in patients having hypotension and poor left ventricular tachyarrhythmia, which includes broad complex tachycardias, or whenever we need an immediate effect in case of highly symptomatic and very unstable patients. In stable patients as well as patients with asthma verapamil is suggested for use, as in these patients a delay of five minutes will not have a negative impact on clinical outcome.

Conditions where it can be safely used are in patients who experienced another episode of SVT briefly after receiving adenosine as they have been experiencing frequent ectopics, which might be ventricular or atrial and could lead to another episode of arrhythmia [17]. Verapamil is suggested for stable patients where a delay of onset of the drug by a few minutes may not result in a worse clinical outcome, and in patients with asthma. Even patients who were previously treated with adenosine and experienced uncomfortable side effects want to avoid adenosine if possible [16].

Etripamil: A Novel Calcium Channel Blocker

Etripamil is a new drug which is recently developed as a “treatment in the pocket” as it can be self-administered intranasally using a spray device by patients who can recognise their symptoms of prolonged PSVT. Etripamil is a rapid-acting, non-dihydropyridine, L-type calcium channel blocker, with a fast onset of action (less than five minutes). It takes about eight minutes on average for peak plasma concentrations of etripamil to be achieved when a 70 mg dose is administered and falls by 60% from a peak value at 25 minutes and becomes 80% within 50 minutes. Therefore, it would be reasonable to anticipate that etripamil will have a direct pharmacological effect on the myocardial calcium current within the first 30 to 40 minutes of administration. Ubiquitous human blood esterase enzymes rapidly inactivate etripamil, resulting in showing its action for a brief period and excellent safety and tolerability profile [8].

Adverse Effects of Both Adenosine and Verapamil

Allergic reaction to concomitant medication; asthenia or pain in arms and legs; chest pain and palpitations are adverse effects of both drugs.

Limitations

As we limited our search to articles in English and have removed grey literature there have been fewer studies to compare the efficacy of adenosine and verapamil. Access to full-text articles was less due to most articles requiring payment for access. 

Acute attack of PSVT being an emergency condition, it is difficult to assess the effectiveness of medications like adenosine and verapamil. To obtain reliable results, we require a prospective, randomised experiment with larger and more precisely distinguished patient populations. Since no patient is on long-term adenosine therapy, it is difficult to assess the efficacy of this drug compared to verapamil in chronic cases of supraventricular tachyarrythmias. This further gives a potential research topic for the future, to carry out prospective cohort studies determining efficacy and safety of long-term adenosine therapy in patients with recurrent PSVT.

## Conclusions

The rate of tachycardia is the main factor that decides which drug would be effective in the treatment of PSVT, and the choice for treatment between the two should be made based on the patient's condition and should be discussed with the patient and reassure the patient in case of adverse effects. verapamil has fewer adverse effects and lasts for a longer duration which might not be desirable in the case of a setting with limited resources, as we are giving the drug through an intravenous route in an emergency setting. Adenosine is the safer option in clinical situations, where verapamil is contraindicated, for example when a patient has hypotension and decreased left ventricular function and is already on treatment of beta‐blockers, or the patient has other tachyarrhythmia such as broad complex tachycardia, or when we need the drug to act rapidly as in very unstable or highly symptomatic patients), side effects are much more common but minor and brief but the onset action is faster compared to verapamil.

If a candidate is suitable for oral CCBs, we recommend using them after the successful termination of an acute PSVT with adenosine. In a setting with very limited resources, CCBs are safe and affordable as compared to adenosine. In this way, prolonged hospital stays can be avoided, reducing further cost of medications, and re-initiating arrhythmia.
